# Enhanced Physical and Mental Function and Promotion of Social Participation in a Cerebral Palsy Patient Through Participation in Para-Jujutsu

**DOI:** 10.7759/cureus.89231

**Published:** 2025-08-01

**Authors:** Tadatoshi Inoue, Shogo Sawamura, Nayu Iwasa, Kato Miu, Lisa Senba

**Affiliations:** 1 Department of Rehabilitation Major in Occupational Therapy, Heisei College of Health Science, Gifu, JPN; 2 Department of Rehabilitation, Heisei College of Health Sciences, Gifu, JPN; 3 Rehabilitation, JA Gifu Kouseiren Seino Kosei Hospital, Gifu, JPN; 4 Rehabilitation Department, Iwasa Hospital Iwasa Maternity, Gifu, JPN; 5 Faculty of Health Sciences, Kumamoto Health Science University, Kumamoto, JPN

**Keywords:** cerebral palsy, jiu-jitsu, occupational therapist, social participation, sports for the disabled

## Abstract

While sports participation for individuals with disabilities is promoted by the Basic Act on Sport and policies for the promotion of parasports, the actual participation rate remains low (32.8% for individuals with disabilities compared to 52.5% for able-bodied individuals), and many challenges exist in continuing competitive sports. While international reports highlight the physical and mental benefits of Jujutsu participation for individuals with disabilities, there are few practical reports on this topic in Japan. This report aims to elucidate, from an occupational therapy perspective, the changes in physical function and quality of life (QOL) experienced by an adult male with cerebral palsy-induced motor paralysis who continuously engaged in Brazilian Jujutsu (BJJ), a combat sport. The subject was a right-handed male in his 30s with motor paralysis in his left upper and lower limbs due to cerebral palsy. He had prior experience in disabled professional wrestling and Judo from age 17, and began BJJ at age 22. We collected initial assessment data (Fugl-Meyer Assessment, Modified Ashworth Scale, range of motion, etc.) and qualitative data based on his narratives, documenting changes gained through BJJ practice and competition participation. During BJJ training, he received technical guidance tailored to his disability characteristics and diligently practiced with an occupational therapist, confirming body movements and techniques. His narratives revealed increased intrinsic motivation, stating things like, “It became easier to move by devising ways to use my left hand and foot”, and “I started wanting to compete in matches”. Twenty years after starting sports, in addition to improvements in physical function (Fugl-Meyer Assessment (FMA) upper extremity 20 points → 59 points, lower extremity 27 points → 47 points, increased range of motion (ROM), and reduced Modified Ashworth Scale (MAS)), the patient experienced an enhanced sense of fulfillment in life due to interpersonal interaction and a sense of achievement in competition. Furthermore, he competed not only in para-jujutsu but also in able-bodied divisions, progressing to the point of winning against able-bodied opponents in the purple belt category. This suggests that the benefits extended beyond mere physical improvement, contributing to the acquisition of psychological fulfillment, such as enhanced self-esteem and the formation of social connections. This case suggests the potential for multifaceted physical, psychological, and social benefits when individuals with disabilities proactively engage in combat sports like BJJ. Moreover, the style of support provided by an occupational therapist who actively participates in the sport alongside the individual is considered effective as a practice that goes beyond mere exercise instruction, closely aligning with the individual’s life and values. Moving forward, it is necessary to accumulate more diverse case studies and research on the potential of disabled sports, including combat sports like Jujutsu, and explore how health support can contribute to the independence and social participation of people with disabilities.

## Introduction

Japan’s Sports Basic Act, enacted in 2011, stipulates that “engaging in a happy and fulfilling life through sports is a right of all people, and all citizens shall be ensured opportunities to routinely enjoy, participate in, or support sports activities under their own initiative, according to their interests, aptitudes, etc., and in a safe and fair environment” [1]. One of its fundamental principles states, “promotion shall be carried out with necessary consideration given to the type and degree of disability, so that persons with disabilities can voluntarily and actively engage in sports” [1]. Around the time of the 2021 Tokyo Paralympics, parasports (sports for people with disabilities) began to gain significant attention in Japan. The Japan Para Sports Association, a public interest incorporated foundation, aims to promote parasports, foster public understanding of disabilities, encourage the independence and social participation of people with disabilities through parasports, and contribute to the realization of a vibrant, inclusive society that respects diversity [2]. Furthermore, parasports encompass sports for all types of disabilities, including not only physical disabilities but also visual, hearing, and intellectual disabilities, and the range of sports is not limited to those featured in the Paralympics. Arakawa [3] identified five challenges in promoting parasports: 1) discovering parasports athletes, 2) securing venues for parasports activities, 3) securing parasports instructors, 4) ensuring the safety of parasports, and 5) improving the competitive performance of parasports.

The participation rate in regular sports among individuals with disabilities is significantly lower at 32.8% compared to 52.5% for individuals without disabilities [4,5]. Moreover, even when medical professionals encourage people with disabilities to participate in sports, the individuals themselves often express reluctance [6]. However, engaging in sports, even with a disability, offers significant physical benefits and has also been shown to influence one’s health perception [7,8]. Overseas studies have reported that participation in para-jujutsu contributes to improvements in physical function and self-esteem for individuals with disabilities, and also positively impacts mental functions. Participation in para-jujutsu has been shown to have both physical and psychological benefits for individuals with disabilities [9,10]. Furthermore, previous research supports the role of systematic physical activity in promoting mental health and social participation in vulnerable populations, including those at risk for depression [11]. However, within Japan, we found no relevant literature when searching “cerebral palsy and jiu-jitsu” in Ichushi (Japan Medical Abstracts Society), Google Scholar, and Medical Online for reports comparing individuals with disabilities before and after participating in Jujutsu, a combat sport.

Therefore, this report describes an occupational therapy approach to a male with cerebral palsy who has long engaged in Jujutsu, utilizing his narrative and physical evaluations. This report aims to examine the significance of Jujutsu for individuals with disabilities and to provide insights for promoting combat sports as a form of parasports in the future.

## Case presentation

The patient is a right-handed male in his 30s with residual motor paralysis in his left upper and lower limbs due to cerebral palsy (Grade 3 disability certificate), which significantly impacts his mobility, balance, and ability to perform bipedal movements. During the day, he works as an office worker performing administrative tasks. In his childhood, the patient played table tennis but found it difficult to perform side steps and backhand returns, which made him realize he was different from others. Subsequently, at the age of 17, he debuted in professional wrestling for people with disabilities, and from ages 18 to 19, he also began practicing wrestling and Judo. Around the age of 22, he started Jujutsu and currently primarily practices and competes in Jujutsu.

Initial evaluation

Table 1 outlines the initial assessment. We assessed the paretic side’s motor function using the Fugl-Meyer Assessment (FMA) [12]. Muscle tone was evaluated with the Modified Ashworth Scale (MAS) [13]. We also measured the range of motion (ROM) in degrees. The initial evaluation data used were from when the patient was 17 years old. His FMA scores were 20 for the upper extremity and 27 for the lower extremity. For the MAS, both upper and lower limbs showed a score of approximately 2. ROM was restricted in both upper and lower limbs. Notably, ROM was measured actively.

**Table 1:** Initial Findings on the Subject’s Physical Function ROM indicates the range of motion for each joint. FMA is an indicator used to evaluate the degree of motor recovery in the paretic limb, with each item scored on a 3-point scale from 0 to 2. A higher score, with a maximum total of 66 points, indicates better motor function. MAS is an indicator used to assess muscle tone, evaluating the degree of muscle resistance to passive movement on a 6-point scale. Higher scores indicate stronger muscle tone and suggest severe spasticity. ROM: range of motion; FMA: Fugl-Meyer Assessment; MAS: Modified Ashworth Scale; MP: metacarpophalangeal; PIP: proximal interphalangeal; DIP: distal interphalangeal

Item	Joint/Area	Movement	ROM (°)
ROM	Shoulder	Flexion	90
		Extension	10
		Abduction	90
		External rotation	10
	Elbow	Extension	-30
		Pronation	-20
	Wrist	Dorsiflexion	0
		Ulnar deviation	10
	Fingers	MP extension	10
		PIP extension	-60
		DIP extension	-30
	Thumb	Palmar abduction	30
		Radial abduction	20
	Hip	Flexion	90
		Extension	0
		Abduction	20
		Adduction	10
		External rotation	10
		Internal rotation	10
	Knee	Extension	-30
	Ankle	Dorsiflexion	-30
FMA	Upper extremity		20
	Lower extremity		27
MAS	Upper extremity		2
	Lower extremity		2

Despite reduced voluntary movement, hypertonia, and restricted ROM, the patient was independent in daily activities by performing tasks within the limits of his paretic side and compensating with his non-paretic side. However, the time required for movements often caused him considerable emotional fatigue due to concerns about inconveniencing others. No significant findings affecting daily life were observed concerning his mental or higher brain functions.

Jujutsu

This report’s primary intervention for the patient was Jujutsu. Prior to the Meiji era, numerous schools of Jujutsu existed in Japan. Jigoro Kano systematically integrated elements from these various schools during the Meiji era to create Kodokan Judo. To popularize Judo worldwide, Mitsuyo Maeda was dispatched from the Kodokan to Brazil, where it evolved into a more practical martial art, which is the modern Jujutsu that is now gaining practitioners globally [14].

Jujutsu is contested one-on-one with a single head referee. The match duration varies by belt color and age, but for the patient’s category, it is 5 minutes. Competitors must wear a clean gi. Jujutsu categories are separated by gender, age, and belt color. Age categories are in 5-year increments, and weight categories are in 6 kg increments. Belts progress incrementally from white (beginner) to blue, purple, brown, and black.

A match is decided by submission or choke. If a competitor clearly taps the opponent, the mat, or themselves twice with their hand, or loses consciousness and faints, it is deemed an “Ippon”. If an Ippon is not achieved within the match time limit, the winner is determined by points. The competitor with the higher score wins: 4 points are awarded for a successful mount position or back control, 3 points for a successful guard pass, and 2 points for a successful takedown, sweep, or knee-on-belly. Fouls include striking, loss of will to fight, and failure to obey the referee’s instructions [15].

Course

The patient realized that practicing Jujutsu served as training for his own body control, so he decided to continue with it. A mentor he met at the Jujutsu dojo provided technical guidance tailored to his disability. Unlike his previous training, he felt his skills had significantly improved under the guidance of his Jujutsu mentor.

The patient recounted, “After starting Jujutsu, I was taught how to use my left leg and left hand. My instructor taught me to use my left elbow like a palm and my left knee like a foot, which made movement much easier. My left oblique muscle is weak, so it’s hard to get up if I’m lying on my right side. If I try to grab my opponent’s right hand to execute a submission, I can’t quite finish it. Other submissions are difficult, but the left side is still easier to use.” These statements indicated his strong motivation toward Jujutsu.

The occupational therapist (OT) participated in Jujutsu practice while listening to the patient’s feedback. The OT had experience in both Judo (black belt) and Jujutsu (purple belt). The OT provided assistance with positioning against opponents, limb placement, center of gravity, and which parts of the opponent’s gi to grasp. Techniques such as takedowns and guard passes were also practiced. The OT regularly engaged in Jujutsu sparring with the patient, reviewing techniques and exchanging ideas regarding body control.

As he continued training, the patient gradually felt a desire to test his skills in competition, leading him to decide to enter Jujutsu matches. From that point on, he became even more focused on his practice. By competing, his challenges became clear, and he began to modify his training content to overcome them. The patient realized that attending the Jujutsu dojo not only improved his body control but also enriched his life through deepening interactions with his dojo mates, including his training partners.

Jujutsu has a Para division specifically for athletes with disabilities, and the patient competed in this division in parallel with the General division for able-bodied competitors. Through repeated competition, he began to win by decision in the Para division.

As the participant’s win rate in the Para division improved, they developed the skill to compete and achieve victories against able-bodied competitors in the purple belt category. The participant expressed feelings such as, “It’s frustrating to lose, even to an able-bodied opponent. I was happy to win”. At 37 years old, the FMA scores were 59 for the upper extremities and 47 for the lower extremities. The MAS was approximately 1 for both upper and lower extremities, and ROM had increased in all areas. The detailed final evaluation is presented in Table 2.

**Table 2:** Results of Each Assessment Item Evaluation Results and Improvement Values for Each Item Before and After Intervention

Item	Joint/Area	Movement	Initial	Final	Difference from Initial
ROM	Shoulder	Flexion	90	180	+80
		Extension	10	35	+25
		Abduction	90	180	+80
		External rotation	10	50	+40
	Elbow	Extension	-30	-10	+20
		Pronation	-20	10	+30
	Wrist	Dorsiflexion	0	20	+20
		Ulnar deviation	10	30	+20
	Fingers	MP extension	10	30	+20
		PIP extension	-60	-20	+40
		DIP extension	-30	-20	+10
	Thumb	Palmar abduction	30	50	+20
		Radial abduction	20	40	+20
	Hip	Flexion	90	100	+10
		Extension	0	15	+15
		Abduction	20	45	+25
		Adduction	10	20	+10
		External rotation	10	30	+20
		Internal rotation	10	30	+20
	Knee	Extension	-30	-10	+20
	Ankle	Dorsiflexion	-30	-5	+25
FMA	Upper Extremity		20	59	+39
	Lower Extremity		27	47	+20
MAS	Upper Extremity		2	1	+1
	Lower Extremity		2	1	+1

Currently, the participant is independent in daily life, though some movements still require more time. Starting Brazilian Jiu-Jitsu not only improved motor function but also fostered new friendships and connections with other disabled athletes competing in para-jujutsu. The establishment of a goal to win matches further enhanced overall life satisfaction. Positive understanding from supervisors and colleagues at work regarding the training environment and competition participation has been secured, and the participant plans to continue competing in the future.

## Discussion

This report highlights the physical and psychological improvements observed in a patient with motor paralysis due to cerebral palsy who engaged in Jujutsu with an OT experienced in the martial art.

Physical improvements

The improvements in FMA score, MAS score, and ROM are likely a result of sustained Jujutsu training, but other factors such as natural recovery and concomitant therapies may also have contributed. Jujutsu demands full-body movements, and the improvements in the paretic upper and lower limbs are presumed to be due to balanced exercise load and increased frequency of muscle and joint usage. Furthermore, the technical guidance in Jujutsu, tailored to his specific disability characteristics, likely contributed to more efficient movements. Specifically, appropriate instruction on body manipulation to compensate for the paretic side is predicted to have led to improved muscle tone and enhanced movement efficiency. In this way, the effective functioning of Jujutsu technical instruction as part of rehabilitation, contributing to strengthening and functional improvement in the paretic side, suggests that Jujutsu may be useful as a form of rehabilitation.

Strength training and stretching exercises are recommended in occupational therapy guidelines [16]. Taylor et al. [17] reported increased muscle strength in individuals with cerebral palsy after 12 weeks of strength training, demonstrating the effectiveness of exercise intervention for this population. Moving forward, it is essential to investigate the effects of Jujutsu in a larger number of cases to explore new possibilities in sports rehabilitation.

Mental and social effects

Through Jujutsu, the patient developed new friendships and set the goal of competing in matches, which led to a profound sense of fulfillment in his overall life. This suggests that the new connections forged through Jujutsu and the goal of competition effectively served as avenues for social participation and self-actualization. Parasports are known to foster confidence and enhance self-esteem through competition, and it appears our patient experienced similar benefits.

Engaging in discussions about his body with the OT during training improved his body control, which, in turn, fueled his desire to test his abilities and ultimately led him to compete in matches. Participating in Jujutsu allowed us to gain new companions and observe an expansion of social interaction. As his winning percentage in the Para division improved, the participant acquired the skills to compete against and defeat able-bodied opponents in the purple belt division. The participant commented, “Even against able-bodied individuals, losing is frustrating. I was happy to win”, “I made new friends by starting Jujutsu and attending the dojo”, “Experiencing Jiu-Jitsu has made my body easier to move, which is helpful in daily life," and "I want to continue competing in the able-bodied division from now on.”

There are reports indicating that Jujutsu practitioners who compete prioritize competitiveness and social recognition more than those who don’t, while non-competitors tend to have a lower correlation with interpersonal relationships as a motivation for engaging in Jujutsu [18]. Our patient, by competing, likely developed a competitive spirit, leading to more creative training methods and increased interactions with others, thus expanding his social horizons. This case clearly demonstrates that parasports contribute not only to physical improvement but also to social participation and mental well-being [19].

Potential and challenges of parasports

The results from this case demonstrate the significant potential of parasports. Specifically, the patient’s participation in a highly competitive sport like Jujutsu and the opportunity to actually compete against able-bodied individuals exemplify social integration through sports. The fact that the patient not only participated in the Para division but also challenged and succeeded in the General (able-bodied) division contributed to his continued engagement in Jujutsu.

On the other hand, a key challenge in promoting parasports is ensuring appropriate environments and qualified instructors [3]. The financial challenges faced by parasports athletes also represent a barrier to parasports promotion [20]. While our patient was employed and financially independent, and experienced dramatic skill improvement through meeting an exceptional instructor, this is not a luxury all parasports athletes can enjoy. To further promote parasports, there is a critical need to establish environments that provide stable income and suitable technical guidance.

Role of occupational therapy

In this report, an OT was involved in the Jujutsu training, contributing to the subject’s body control assessment and technical skill improvement. Given Jujutsu’s competitive nature, the patient’s ability to learn efficient movements was likely due to the OT’s specific guidance on crucial elements such as positioning relative to the opponent, limb usage, and managing the center of gravity.

A survey of Jujutsu practitioners reported that interest, enjoyment, and skill improvement were high training motivations, regardless of years of experience [21]. Additionally, Jujutsu has been reported to have a low incidence of injuries requiring surgical intervention [22]. Moving forward, it will be essential for OTs to continue their active involvement in guiding parasports athletes, providing both physical and psychological support.

Limitations

A limitation of this study is that it focuses on a single case of a male in his 30s with cerebral palsy, making it difficult to generalize the results to other individuals with disabilities or those from different backgrounds. To demonstrate the effectiveness for a broader population of people with disabilities, larger-scale studies involving a more diverse range of subjects are necessary. Additionally, while the Jujutsu intervention contributed to physical and mental improvements, the specific mechanisms by which Jujutsu’s characteristics or other factors precisely exerted their effects are not entirely clear. Future research should aim to increase the number of cases to further investigate the effects of Jujutsu.

## Conclusions

This case report details a patient who engaged in a Jujutsu-centered intervention over a long period of 20 years. The results obtained are attributed to an occupational therapist with Jujutsu experience who sparred with the patient, aiming to improve both physical function and Jujutsu performance. As his Jujutsu skills improved, the patient gained new goals through successful experiences, such as defeating able-bodied opponents, and by forming connections with training partners. This suggests that Jujutsu may contribute to the improvement of physical function and quality of life for individuals with cerebral palsy.
